# A novel dynamic Bayesian network approach for data mining and survival data analysis

**DOI:** 10.1186/s12911-022-02000-7

**Published:** 2022-09-22

**Authors:** Ali Sheidaei, Abbas Rahimi Foroushani, Kimiya Gohari, Hojjat Zeraati

**Affiliations:** 1 Department of Epidemiology and Biostatistics, School of Public Health, Tehran University of Medical Sciences, Pour Sina St., Keshavarz Blvd., Tehran, 14176-13151 Iran; 2 Department of Biostatistics, Faculty of Medical Sciences, Tarbiat Modares University, Tehran, Iran

**Keywords:** Dynamic Bayesian network, Directed acyclic graph, Survival analysis, Gastric cancer

## Abstract

**Background:**

Censorship is the primary challenge in survival modeling, especially in human health studies. The classical methods have been limited by applications like Kaplan–Meier or restricted assumptions like the Cox regression model. On the other hand, Machine learning algorithms commonly rely on the high dimensionality of data and ignore the censorship attribute. In addition, these algorithms are more sophisticated to understand and utilize. We propose a novel approach based on the Bayesian network to address these issues.

**Methods:**

We proposed a two-slice temporal Bayesian network model for the survival data, introducing the survival and censorship status in each observed time as the dynamic states. A score-based algorithm learned the structure of the directed acyclic graph. The likelihood approach conducted parameter learning. We conducted a simulation study to assess the performance of our model in comparison with the Kaplan–Meier and Cox proportional hazard regression. We defined various scenarios according to the sample size, censoring rate, and shapes of survival and censoring distributions across time. Finally, we fit the model on a real-world dataset that includes 760 post gastrectomy surgery due to gastric cancer. The validation of the model was explored using the hold-out technique based on the posterior classification error. Our survival model performance results were compared using the Kaplan–Meier and Cox proportional hazard models.

**Results:**

The simulation study shows the superiority of DBN in bias reduction for many scenarios compared with Cox regression and Kaplan–Meier, especially in the late survival times. In the real-world data, the structure of the dynamic Bayesian network model satisfied the finding from Kaplan–Meier and Cox regression classical approaches. The posterior classification error found from the validation technique did not exceed 0.04, representing that our network predicted the state variables with more than 96% accuracy.

**Conclusions:**

Our proposed dynamic Bayesian network model could be used as a data mining technique in the context of survival data analysis. The advantages of this approach are feature selection ability, straightforward interpretation, handling of high-dimensional data, and few assumptions.

**Supplementary Information:**

The online version contains supplementary material available at 10.1186/s12911-022-02000-7.

## Background

Survival analysis is the formal name for an essential branch of statistics. It aims to study the time of the specific event and explore the particular circumstances or characteristics that influence it [1]. We know these techniques as reliability analysis in engineering and industries. The usual interest is the lifetime of devices, products, and machines in these contexts [2–4]. The intrinsic features of subjects in these domains enable the investigators to control all the processes and fully observe the data [3, 5].

Further, when the subjects are humans, the survival models confront the crucial challenge of incomplete data and the high dimension of confounders more than the other context. The incomplete data known as censoring is expected because sample withdrawal or loss of follow-up exceeds the event time of the study period [6, 7]. Despite all efforts to evolve the survival models, some challenges in classical methods show the necessity of developing new approaches in practice [8–10].

The Kaplan–Meier method, also known as product-limit, is one of the primary and still popular methods in analyzing survival data. This method estimated the survival probability function of data in a straightforward and easy-to-understand manner. In addition, the Kaplan–Meier is a nonparametric approach, and a few assumptions are required to apply it to data [1, 11]. However, the simplicity of Kaplan–Meier restricts its applications. For instance, it cannot account for multiple factors or control for confounding factors. Therefore, in the case of feature selection and group comparisons, we need to apply additional analyses such as the low power log-rank test. In this procedure, insufficient sample size and increasing the number of features lead to inaccurate inferences [12]. Another alternative is regression approaches like the Cox proportional hazard model. Using these models is limited by restrictive assumptions like the proportionality of hazards, independence of censoring and survival distribution, and exponential relation between hazard and covariates [13].

On the other hand, developing the application of machine learning algorithms in recent decades has reformed many classical approaches to data analysis. The survival analysis is one of the domains that changed significantly [2, 9, 10]. In this manner, the applications of random forests [14, 15], Bayesian methods [5, 16, 17], neural networks [18–21], support vector machines [22, 23], ensemble learning [24, 25], and active learning [26, 27] algorithms were introduced in survival analysis. These changes enable us to resolve issues with a new practice even though the general idea is similar to classical approaches.

As mentioned, incompletely observed data or censorship is the primary challenge in survival modeling, especially in health. Considerable studies that introduced the application of machine learning algorithms in survival analysis did not engage the censorship because of the study subject's type [5]. Some other studies relied on high sample size and ignored the censored observations or imputed them by modeling approaches [28, 29]. Finally, a few studies directly address censorship using the methods like weighting the censored observation [30]. However, there is a gap between classical approaches and novel machine learning techniques that use intelligent algorithms to extract data patterns. The classic methods were developed to handle small to medium-dimension data and find a general overview. In contrast, the machine learning algorithm and data mining techniques aim to handle high-dimensional data. These methods focus on the prediction with maximum accuracy.

This study proposes a novel Dynamic Bayesian Network (DBN) model for data mining in the context of survival data analysis. The Bayesian Network (BN) has a series of powerful tools that could facilitate survival analysis. Actually, the BN combines probability theory and graphical models [31]. Consequently, it enabled us to capture the uncertainty of stochastic survival events and represent a graphical structure of probability distribution. In addition, our model uses the Kaplan–Meier idea to consider the censoring phenomena and the various capability of BN models to add extra tools for more precise inferences simultaneously. The structure learning algorithm of the BN ables us to compare the groups and find the significant features. In addition, parameter learning algorithms lead to more precise inferences, estimations, and predictions. In this study, we present our DBN model for survival analysis, evaluate its performance using a simulation study, and finally use a real-world data set to show the way analysis could be performed using that.

## Methods

### Product limit estimators

The primary objective of survival analysis is to explore the time until a particular event. Hence, we describe the stochastic behavior of an outcome variable in time type. We usually use survival, density, hazard, and the mean or median residual life functions in this regard. As these functions are attainable from each other, there is no priority except for better interpretability in choosing one. The product limit estimators are the estimates of survival function, which is defined as the probability of an individual surviving after a given time point t:

$$S\left( t \right) = {\text{P}}(T > t)$$

T is a random variable that denotes when the event of interest occurs. Kaplan and Meier partitioned observed times into intervals according to unique event times and proposed the following estimator for all t values in the range of observed data when $t_{1}$ represent the first event time [1]:

1 $$\hat{S}\left( t \right) = \left\{ {\begin{array}{*{20}l} 1 \hfill & {if\;t < t_{1} } \hfill \\ {\mathop \prod \limits_{{t_{i} \le t}} \left[ {1 - \frac{{d_{i} }}{{Y_{i} }}} \right] } \hfill & {if\;t_{1} \le t} \hfill \\ \end{array} } \right.$$

where $d_{i}$ and $Y_{i}$ represent the number of failures and at-risk persons in each interval, respectively. Therefore, the product-limit estimator is a discrete approach that leads to a step function that only changes at event times. The Greenwood formula is the well-known approach to estimating the variance of the estimators [1]:

2 $$\hat{V}\left[ {\hat{S}\left( t \right)} \right] = \hat{S}\left( t \right)^{2} \mathop \sum \limits_{{t_{i} \le t}} \frac{{d_{i} }}{{Y_{i} \left( {Y_{i} - d_{i} } \right)}}$$

### Bayesian network

Every Bayesian Networks (BNs) correspond to a Directed Acyclic Graph (DAG) and a joint distribution, which are the graphical and probabilistic aspects of the model. DAG consists of nodes corresponding to random variables and edges that present conditional probabilities. According to the domain of the random variables, the BN could be discrete, Gaussian, or hybrid. Our BN in this study is a discrete one. Therefore, for a set of discrete random variables ${\varvec{X}} = \left( {X_{1} ,X_{2} , \ldots ,X_{D} } \right){ }$ Taking their values in the discrete and finite D-dimensional domain. The BN is defined as pair ${\mathcal{M}} = \left( {{{\mathcal{g}}},\left( {P\left( {X_{d} |{\mathbf{\mathcal{P}}}_{{\varvec{X}}} \left( {X_{d} } \right)} \right)} \right)_{1 \le d \le D} } \right)$ where ${{\mathcal{g}}} = \left( {{\varvec{X}},{\varvec{\varepsilon}}} \right)$ is a DAG presentation of random variables ${\varvec{X}}$ with edges set ${\varvec{\varepsilon}}$, ${\mathbf{\mathcal{P}}}_{{\varvec{X}}} \left( {X_{d} } \right)$ is the set of parents $X_{d}$ in ${\varvec{X}}$, and $\left( {P\left( {X_{d} |{\mathbf{\mathcal{P}}}_{{\varvec{X}}} \left( {X_{d} } \right)} \right)} \right)_{1 \le d \le D}$ is the conditional probability of node $X_{d}$ given their parents in the set ${\varvec{X}}$.

The appealing feature of BN is to summarize the complex joint probability distribution ${\varvec{X}}$ in the following parsimonious way using the conditional independence and Markov chain properties:

3 $$P\left( {X_{1} ,X_{2} , \ldots ,X_{D} } \right) = \mathop \prod \limits_{d = 1}^{D} P\left( {X_{d} |{\mathbf{\mathcal{P}}}_{{\varvec{X}}} \left( {X_{d} } \right)} \right)$$

### Dynamic Bayesian network

The classical BN is not adopted to address time-dependent processes like survival analysis [32]. Therefore, Dynamic Bayesian Network (DBN) [33] was introduced to extend this process. In this context, time-dependent random variables $\left( {{\varvec{X}}_{t} } \right)_{t \ge 1} = \left( {X_{1,t} , \ldots ,X_{D,t} } \right)_{t \ge 1}$ are defined where *t* is a discrete index time formally called slice. DBN uses Markov property which indicates the future of a stochastic process is independent of its past, given current status or several lags before it. The number of lags determines the order of the Markov process. This study only needs to use the Markov process of order 1, which leads to a 2-slice Temporal Bayesian Network (2-TBN). In this regard, we assume $X_{t - 1} \bot X_{t + 1} |X_{t}$ for all $t \ge 2$. A 2-TBN could be defined as a pair of 2 BNs $\left( {{\mathcal{M}}_{1} ,{\mathcal{M}}_{ \to } } \right)$ where ${\mathcal{M}}_{1}$ is the joint distribution of the initial process ${\varvec{X}}_{1} = \left( {X_{1,1} , \ldots ,X_{D,1} } \right)$ and ${\mathcal{M}}_{ \to }$ represent the transition model. The joint probability distribution ${\mathcal{M}}_{1}$ easily derived from BN approach in Eq. 3:

4 $$P\left( {{\varvec{X}}_{1} } \right) = \mathop \prod \limits_{d = 1}^{D} P\left( {X_{d,1} |{\mathbf{\mathcal{P}}}_{{\varvec{X}}} \left( {X_{d,1} } \right)} \right)$$

In the transition model, the joint distribution of ${\varvec{X}}_{t}$ only depends on random variables belonging to the set of parents ${\varvec{X}}_{t}$ at slice $t - 1$ in the form:

5 $$P\left( {{\varvec{X}}_{t} {|}{\varvec{X}}_{t - 1} } \right) = P\left( {X_{1,t} , \ldots ,X_{D,t} {|}X_{1,t - 1} , \ldots ,X_{D,t - 1} } \right) = \mathop \prod \limits_{d = 1}^{D} P\left( {X_{d,t} |{\mathbf{\mathcal{P}}}_{{{\varvec{X}}_{t} }} \left( {X_{d,t} } \right)} \right)$$

Hence the probability distribution of 2-TBN is calculated by the combination of Eqs. 4,5:

6 $$P\left( {{\varvec{X}}_{1 \le t \le T} } \right) = \mathop \prod \limits_{d = 1}^{D} P(X_{d,1} |{\mathbf{\mathcal{P}}}_{{\varvec{X}}} \left( {X_{d,1} } \right))\mathop \prod \limits_{t = 2}^{T} \mathop \prod \limits_{d = 1}^{D} P\left( {X_{d,t} |{\mathbf{\mathcal{P}}}_{{{\varvec{X}}_{t} }} \left( {X_{d,t} } \right)} \right)$$

In order to consider the time stationary covariates ${\varvec{Z}} = \left( {Z_{1} , \ldots ,Z_{q} } \right)$ in the model, we could extend the parent sets in both initial processes ${\mathcal{M}}_{1}$ and transition model ${\mathcal{M}}_{ \to }$. In this manner, the initial conditional probability could be presented as:

$$P\left( {{\varvec{X}}_{1} } \right) = \mathop \prod \limits_{d = 1}^{D} P\left( {X_{d,1} |{\mathbf{\mathcal{P}}}_{{{\varvec{X}}_{t} }} \left( {X_{d,t} } \right),{\mathbf{\mathcal{P}}}_{{\varvec{Z}}} \left( {X_{d,1} } \right)} \right)$$

where the ${\mathbf{\mathcal{P}}}_{{\varvec{X}}} \left( {X_{d,1} } \right)$ and ${\mathbf{\mathcal{P}}}_{{\varvec{Z}}} \left( {X_{d,1} } \right)$ represent the sets of parents $X_{d,1}$ in ${\varvec{X}}$ and ${\varvec{Z}},$ respectively. On the other hand, the modified transition probability distribution is reformed to:

$$P\left( {{\varvec{X}}_{t} {|}{\varvec{X}}_{t - 1} ,Z_{1} , \ldots ,Z_{q } } \right) = P\left( {X_{1,t} , \ldots ,X_{D,t} {|}X_{1,t - 1} , \ldots ,X_{D,t - 1} ,Z_{1} , \ldots ,Z_{q} } \right) = \mathop \prod \limits_{d = 1}^{D} P\left( {X_{d,t} |{\mathbf{\mathcal{P}}}_{{{\varvec{X}}_{t} }} \left( {X_{d,t} } \right),{\mathbf{\mathcal{P}}}_{{\varvec{Z}}} \left( {X_{d,t} } \right)} \right)$$

### Dynamic Bayesian network interpretation of product limit estimators

**Fig. 1:**
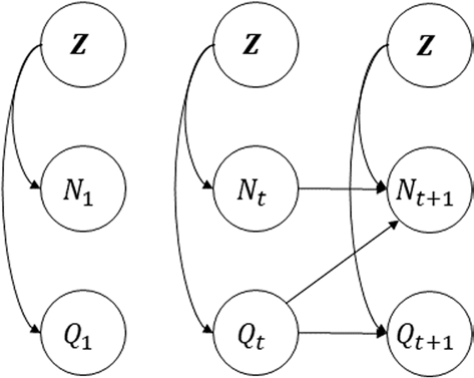
Prior and transition Bayesian networks correspond to the extended dynamic Bayesian network representation of the Kaplan–Meier approach

A DBN of type 2-TBN could efficiently conduct the calculation process of product-limit estimators. The product-limit approach considers the time as discrete intervals between consecutive observed failure times and counts the individuals at risk and failures. Equivalently, we define discrete intervals of Kaplan–Meier as slices and two time-dependent binary status variables. The survival state variables $N_{i,t}$ is equal to 1 if individual $i$ survives at least to slice t and state variables $Q_{i,t}$ is equal to 1 if the individual $i$ censored before or at slice t. Defining these variables enables us to form the DBN in Fig. 1 to analyze survival data. In addition, we can enter other fixed effect covariates in ${\varvec{Z}}$ to the model and examine their importance by the structure learning algorithms.

The DBN estimators of survival probability at each time t according to Eq. 6 are equal to:

7 $$\hat{S}\left( t \right) = P\left( {N_{1} = 1|{\mathbf{\mathcal{P}}}_{{\varvec{Z}}} \left( {N_{t} } \right)} \right)\mathop \prod \limits_{t = 2}^{T} P\left( {N_{t} = 1|N_{t - 1} = 1,Q_{t - 1} = 0,{\mathbf{\mathcal{P}}}_{{\varvec{Z}}} \left( {N_{t} } \right)} \right)$$

Moreover, the discrete covariates set ${\mathbf{\mathcal{P}}}_{{\varvec{Z}}} \left( {N_{t} } \right)$ were found by structure learning algorithms.

For simplicity, we consider there are no covariates in the model, so Eq. (7) could be modified by counter function N:

8 $$\begin{aligned} \hat{S}\left( t \right) = P\left( {N_{1} = 1} \right)\mathop \prod \limits_{t = 2}^{T} P\left( {N_{t} = 1{|}N_{t - 1} = 1,Q_{t - 1} = 0} \right) = & \frac{{N\left( {N_{i1} = 1} \right)}}{N}\mathop \prod \limits_{t = 2}^{T} \frac{{N\left( {N_{it} = 1} \right)}}{{N\left( {N_{i,t - 1} = 1, Q_{i,t - 1} = 0} \right)}} \\ = & \hat{S}\left( 1 \right)\mathop \prod \limits_{t = 2}^{T} \left[ {1 - \frac{{d_{t} }}{{Y_{t} }}} \right] \\ \end{aligned}$$

That is the same as product-limit estimators in Eq. (1). Therefore, we are able to use the Greenwood formula in Eq. (2) to calculate the variance of survival estimations. In addition, the common bootstrapping approaches in the BN context, like likelihood weighting and logic sampling, are the alternative approach in this way[34].

### Simulation study

We conducted a simulation study to assess the performance of our model in comparison with the Kaplan–Meier and Cox proportional hazard regression. We defined various scenarios according to the sample size (N = 800, 5000, 10,000), censoring rate (R = 25%, 40%, 60%), and shape parameters of survival ($\alpha_{S}$) and censoring ($\alpha_{C}$) distributions. We considered five covariates distributed as mutually independent binomial distributions with different probability parameters $\left[ {X_{i} \sim B\left( {N,P_{i} } \right), i = 1, \ldots ,5 P_{i} = 0.1, 0.2, 0.5, 0.7, 0.9} \right]$.

The survival and censoring times were generated using Weibull distributions. The scale parameter of survival time distribution was reparametrized according to the summation of the covariates $\left[ {\theta_{S} = \mathop \sum \limits_{i = 1}^{5} X_{i} } \right]$. Using the numerical methods and assuming the fixed value for censorship, we found the scale parameter of censoring time distribution. We set the shape parameter of survival and censoring distributions as the values 0.5 (decreasing event/censor rate), 1 (constant event/censor rate), and 2 (increasing event/censor rate). In this manner, we achieved nine different scenarios for the shape of survival/censoring times.

In brief, if the survival time $S \ge 0$ follows the below Weibull distribution:

$$f\left( {s;\alpha_{S} ,\theta_{S} } \right) = \frac{{\alpha_{S} }}{{\theta_{S} }}\left( {\frac{s}{{\theta_{S} }}} \right)^{{\alpha_{S} - 1}} {\text{exp}}\left( { - \left( {\frac{s}{{\theta_{S} }}} \right)^{{\alpha_{S} }} } \right)$$

where scale parameter $\theta_{S}$ and shape parameter $\alpha_{S}$ were defined before, the cumulative distribution function of S is:

$$F\left( {s;\alpha_{S} ,\theta_{S} } \right) = 1 - {\text{exp}}\left( { - \left( {\frac{s}{{\theta_{S} }}} \right)^{{\alpha_{S} }} } \right)$$

As the Weibull is a continuous random distribution $$F\left( {s;\alpha_{S} ,\theta_{S} } \right)\sim U\left( {0,1} \right)$$. Therefore we generated N samples $$u_{i} \sim U\left( {0,1} \right)$$ for $$i = 1, \ldots ,N$$ and then compute the $$s_{i} = \theta_{S} \sqrt[{{\raise0.7ex\hbox{$1$} \!\mathord{\left/ {\vphantom {1 {\alpha_{S} }}}\right.\kern-\nulldelimiterspace} \!\lower0.7ex\hbox{${\alpha_{S} }$}}}]{{ - {\text{ln}}\left( {u_{i} } \right)}}$$. A similar approach was used to generate the censored times $$c_{i} s$$.

We fitted all three models to the simulated data and estimated survival probability at 20%, 50% (median), and 80% percentiles of actual survival times. The bias and its Root Mean Squared Errors (RMSE) were calculated in 1000 randomly generated samples in each scenario.

### Real data analysis

We applied our proposed method to real-world survival data. The 760 patients diagnosed with gastric cancer at the Iran cancer Institute and who had undergone gastrectomy from 1995 to 2012 entered the study. This historical and artificial cohort of patients was followed until observing death events. The censorship was considered in case of loss to follow-up or being alive at the end of observation time. All the variables except follow-up duration were time stationery and were collected at the surgery time.

We conducted the ordinary Kaplan–Meier survival analysis, DBN, and Cox proportional hazard (PH) model. The Cox PH model was added to compare our findings with a regression model that could handle the covariate effect in survival analysis. In addition, the censor probability plots were generated using the Kaplan–Meier approach. For this purpose, we defined censorship as the primary event and death as the alternative status.

### Structure learning

We used Hill-Climbing (HC) and Tabu search, two of the most popular score-based learning algorithms, to find the structure of DAG [35]. The last event occurred 15 years after surgery, and there was no event in year 13 post-surgery. Therefore 14 eligible slices $t$ correspond to the unique event year were defined in the DBN structure. The prior and transition networks in Fig. 1 related to $N_{i,t}$ and $Q_{i,t}$ were considered as the white list, and the algorithms learned the structure of stationary variables Z. The validation of structure learning was conducted by the ten repeated hold-out technique using ten subsamples in size 30% of the original data [36]. The posterior classification error based on likelihood weighting was set as the loss function [37]. The validation process was repeated according to different score functions, including logarithm of likelihood, AIC, BIC, BDE, BDS, and $K^{2}$ [38].

## Results

### Simulation study

Table 1 represents the bias and related RMSE of estimated survival probability by models in different scenarios. For instance, the first value of − 0.0033 (0.0046) is observed for bias (RMSE) for a sample size of 800 and censorship of 25%. It shows that the Kaplan–Meier approach estimated the survival probability by 0.0033 less than the actual value when the random samples come from the survival and censoring distributions with increasing rates over time. Cox and DBN estimations in this scenario are 0.004 and − 0.0008 differ from the actual probability, respectively. Therefore, the minimum absolute bias value is related to the DBN model.

**Table 1:** The models' bias and RMSE in survival probability estimation in percentile 20% of real survival time

*α*_S_	R(%)	N	*α*_C_ = 2			*α*_C_ = 1			*α*_C_ = 0.5		
			KM	Cox	DBN	KM	Cox	DBN	KM	Cox	DBN
2	25	800	− 0.0033 (0.0046)	0.004 (0.0056)	− 0.0008 (0.0063)	− 0.0081 (0.0099)	0.0005 (0.0059)	− 0.0054 (0.0093)	− 0.0122 (0.0145)	− 0.0026 (0.0079)	− 0.0088 (0.0129)
2	25	5000	− 0.0035 (0.0037)	0.0045 (0.0048)	− 0.0022 (0.0046)	− 0.008 (0.0083)	0.0011 (0.0025)	− 0.0061 (0.0077)	− 0.0123 (0.0127)	− 0.0018 (0.0035)	− 0.0093 (0.0109)
2	25	10,000	− 0.0035 (0.0036)	0.0046 (0.0047)	− 0.0029 (0.0049)	− 0.0081 (0.0083)	0.0011 (0.0019)	− 0.0065 (0.0078)	− 0.0123 (0.0125)	− 0.0018 (0.0027)	− 0.0094 (0.0105)
2	40	800	− 0.0073 (0.0088)	− 0.0006 (0.0052)	− 0.005 (0.0088)	− 0.0144 (0.0165)	− 0.0061 (0.01)	− 0.0112 (0.0149)	− 0.0212 (0.0235)	− 0.0111 (0.0147)	− 0.0171 (0.0205)
2	40	5000	− 0.0077 (0.008)	− 0.0004 (0.0021)	− 0.006 (0.0075)	− 0.0145 (0.0149)	− 0.0055 (0.0063)	− 0.0113 (0.0125)	− 0.0204 (0.0208)	− 0.0094 (0.0101)	− 0.016 (0.0172)
2	40	10,000	− 0.0077 (0.0078)	− 0.0004 (0.0015)	− 0.0066 (0.0077)	− 0.0146 (0.0147)	− 0.0055 (0.0059)	− 0.0116 (0.0125)	− 0.0206 (0.0207)	− 0.0095 (0.0099)	− 0.0159 (0.0168)
2	60	800	− 0.0174 (0.0192)	− 0.0122 (0.0144)	− 0.0151 (0.0176)	− 0.0278 (0.0299)	− 0.0195 (0.0223)	− 0.024 (0.027)	− 0.0353 (0.0382)	− 0.0242 (0.028)	− 0.0315 (0.0353)
2	60	5000	− 0.0185 (0.0188)	− 0.0123 (0.0127)	− 0.0158 (0.0167)	− 0.0282 (0.0285)	− 0.0191 (0.0196)	− 0.0231 (0.0241)	− 0.0359 (0.0363)	− 0.0239 (0.0245)	− 0.0296 (0.0307)
2	60	10,000	− 0.0186 (0.0187)	− 0.0123 (0.0124)	− 0.0157 (0.0164)	− 0.0281 (0.0283)	− 0.0189 (0.0191)	− 0.0223 (0.023)	− 0.0355 (0.0357)	− 0.0233 (0.0237)	− 0.0282 (0.029)
1	25	800	− 0.0012 (0.002)	0.0016 (0.0028)	0.0001 (0.005)	− 0.0029 (0.0039)	− 0.0007 (0.003)	− 0.0023 (0.0058)	− 0.0075 (0.0088)	− 0.0052 (0.0071)	− 0.0065 (0.009)
1	25	5000	− 0.0011 (0.0013)	0.002 (0.0022)	− 0.0006 (0.0046)	− 0.0028 (0.003)	− 0.0005 (0.0012)	− 0.0029 (0.0051)	− 0.0075 (0.0077)	− 0.0051 (0.0054)	− 0.0074 (0.0086)
1	25	10,000	− 0.0011 (0.0012)	0.0021 (0.0022)	− 0.0009 (0.004)	− 0.0028 (0.0029)	− 0.0004 (0.0009)	− 0.0027 (0.0049)	− 0.0076 (0.0077)	− 0.0051 (0.0053)	− 0.0075 (0.0086)
1	40	800	− 0.0031 (0.0041)	− 0.0004 (0.0031)	− 0.0018 (0.0058)	− 0.0057 (0.0067)	− 0.0036 (0.0052)	− 0.0047 (0.0074)	− 0.0137 (0.0151)	− 0.0114 (0.013)	− 0.0128 (0.015)
1	40	5000	− 0.0031 (0.0033)	− 0.0002 (0.0012)	− 0.0027 (0.0054)	− 0.0058 (0.006)	− 0.0035 (0.0038)	− 0.0054 (0.0069)	− 0.014 (0.0142)	− 0.0115 (0.0118)	− 0.0135 (0.0144)
1	40	10,000	− 0.0031 (0.0032)	− 0.0001 (0.0009)	− 0.0026 (0.005)	− 0.0058 (0.0059)	− 0.0034 (0.0036)	− 0.0056 (0.0069)	− 0.0139 (0.014)	− 0.0114 (0.0115)	− 0.0136 (0.0143)
1	60	800	− 0.0043 (0.005)	− 0.0023 (0.0037)	− 0.0036 (0.006)	− 0.0135 (0.0146)	− 0.0113 (0.0127)	− 0.0125 (0.0144)	− 0.0256 (0.027)	− 0.0231 (0.0247)	− 0.0243 (0.0261)
1	60	5000	− 0.0045 (0.0048)	− 0.0023 (0.0028)	− 0.0042 (0.006)	− 0.0132 (0.0134)	− 0.0109 (0.0112)	− 0.0129 (0.0137)	− 0.0255 (0.0257)	− 0.0229 (0.0232)	− 0.0246 (0.0251)
1	60	10,000	− 0.0047 (0.0051)	− 0.0025 (0.0031)	− 0.0045 (0.0063)	− 0.0131 (0.0132)	− 0.0108 (0.0109)	− 0.0129 (0.0136)	− 0.0256 (0.0257)	− 0.0229 (0.0231)	− 0.0251 (0.0256)
0.5	0.25	800	0 (0.0001)	0.001 (0.0013)	0.0005 (0.0044)	− 0.0004 (0.001)	0.0006 (0.0013)	0.0004 (0.0044)	− 0.0029 (0.0039)	− 0.0019 (0.0032)	− 0.0018 (0.0056)
0.5	25	5000	0 (0)	0.0011 (0.0011)	0.0005 (0.0039)	− 0.0005 (0.0006)	0.0007 (0.0008)	− 0.0002 (0.0042)	− 0.0031 (0.0033)	− 0.0019 (0.0023)	− 0.0027 (0.0049)
0.5	25	10,000	0 (0)	0.0011 (0.0011)	0.0002 (0.0042)	− 0.0005 (0.0005)	0.0007 (0.0007)	− 0.0005 (0.0042)	− 0.0031 (0.0032)	− 0.0019 (0.0021)	− 0.0031 (0.0052)
0.5	40	800	− 0.0001 (0.0003)	0.001 (0.0013)	0.0008 (0.0045)	− 0.0011 (0.0018)	− 0.0001 (0.0016)	− 0.0007 (0.0047)	− 0.0061 (0.0072)	− 0.005 (0.0064)	− 0.0056 (0.008)
0.5	40	5000	0 (0.0001)	0.0011 (0.0011)	− 0.0001 (0.0041)	− 0.0013 (0.0014)	− 0.0001 (0.0007)	− 0.001 (0.0043)	− 0.006 (0.0062)	− 0.0049 (0.0051)	− 0.0058 (0.0073)
0.5	40	10,000	0 (0.0001)	0.0011 (0.0011)	0.0001 (0.004)	− 0.0012 (0.0013)	− 0.0001 (0.0005)	− 0.0009 (0.0041)	− 0.006 (0.0061)	− 0.0049 (0.005)	− 0.0059 (0.0072)
0.5	60	800	− 0.0007 (0.0012)	0.0004 (0.0013)	− 0.0001 (0.0046)	− 0.0045 (0.0053)	− 0.0034 (0.0044)	− 0.0038 (0.0064)	− 0.0136 (0.0147)	− 0.0125 (0.0137)	− 0.0128 (0.0145)
0.5	60	5000	− 0.0007 (0.0008)	0.0005 (0.0007)	− 0.0005 (0.004)	− 0.0046 (0.0048)	− 0.0035 (0.0037)	− 0.0045 (0.0063)	− 0.0138 (0.014)	− 0.0126 (0.0128)	− 0.0134 (0.0142)
0.5	60	10,000	− 0.0007 (0.0008)	0.0005 (0.0006)	− 0.0006 (0.004)	− 0.0046 (0.0047)	− 0.0034 (0.0035)	− 0.0043 (0.0061)	− 0.0136 (0.0137)	− 0.0124 (0.0125)	− 0.0135 (0.0142)

The Cox regression model shows superiority according to the minimum bias for most scenarios in Table 1. On the other hand, the DBN estimates actual survival probability better than the Cox approach in 52% of simulation scenarios which assumes an increasing censor rate across time. Increasing the censoring rate causes higher observed bias (RMSE) for all the models. The constant censor rate across time (alpha c = 1), which corresponds to non-informative censoring, shows lower levels of bias (RMSE) in all the scenarios.

Similar results for exploring median survival time are presented in Table 2. The number of scenarios in which the DBN is superior to other models due to bias (RMSE) reduction is relatively higher than in Table 1. In addition, Cox is not significantly better than DBN in all situations, and in many cases, its absolute bias is less than 0.001 of DBN.

**Table 2:** The models' bias and RMSE in survival probability estimation in the median of real survival time

*α*_S_	R(%)	N	*α*_C_ = 2			*α*_C_ = 1			*α*_C_ = 0.5		
			KM	Cox	DBN	KM	Cox	DBN	KM	Cox	DBN
2	25	800	− 0.0104 (0.0125)	− 0.0043 (0.0082)	− 0.0037 (0.0102)	− 0.0105 (0.0135)	− 0.0022 (0.0084)	− 0.0035 (0.0112)	− 0.0107 (0.0143)	− 0.0001 (0.0089)	− 0.0028 (0.012)
2	25	5000	− 0.01 (0.0104)	− 0.0033 (0.0043)	− 0.0057 (0.0079)	− 0.0105 (0.011)	− 0.001 (0.0032)	− 0.0049 (0.0078)	− 0.0106 (0.0112)	0.0009 (0.0036)	− 0.0039 (0.0077)
2	25	10,000	− 0.0098 (0.01)	− 0.0029 (0.0035)	− 0.0058 (0.0076)	− 0.0106 (0.0109)	− 0.0011 (0.0025)	− 0.0056 (0.0077)	− 0.0106 (0.011)	0.001 (0.0027)	− 0.005 (0.0075)
2	40	800	− 0.0211 (0.0235)	− 0.0158 (0.0185)	− 0.0144 (0.019)	− 0.0195 (0.0229)	− 0.0114 (0.0161)	− 0.0117 (0.018)	− 0.0178 (0.0227)	− 0.0069 (0.0139)	− 0.0097 (0.0184)
2	40	5000	− 0.021 (0.0215)	− 0.0154 (0.0159)	− 0.0145 (0.0159)	− 0.0192 (0.0198)	− 0.0104 (0.0113)	− 0.0113 (0.0134)	− 0.0181 (0.0189)	− 0.0063 (0.0078)	− 0.0095 (0.0125)
2	40	10,000	− 0.0211 (0.0213)	− 0.0154 (0.0157)	− 0.0148 (0.0158)	− 0.0196 (0.0198)	− 0.0108 (0.0112)	− 0.0115 (0.0129)	− 0.0181 (0.0185)	− 0.0062 (0.0071)	− 0.01 (0.0119)
2	60	800	− 0.0368 (0.0399)	− 0.0302 (0.0335)	− 0.0312 (0.0354)	− 0.0379 (0.0426)	− 0.0298 (0.0346)	− 0.0306 (0.0374)	− 0.0322 (0.0384)	− 0.0217 (0.0282)	− 0.0234 (0.0326)
2	60	5000	− 0.0434 (0.0443)	− 0.0367 (0.0379)	− 0.0344 (0.036)	− 0.0379 (0.0387)	− 0.029 (0.0298)	− 0.0255 (0.0275)	− 0.0321 (0.0332)	− 0.02 (0.0213)	− 0.0198 (0.0228)
2	60	10,000	− 0.046 (0.0466)	− 0.0394 (0.0403)	− 0.0348 (0.0358)	− 0.0378 (0.0382)	− 0.0288 (0.0292)	− 0.024 (0.0252)	− 0.0314 (0.0319)	− 0.0191 (0.0198)	− 0.0176 (0.0196)
1	25	800	− 0.008 (0.0095)	− 0.0065 (0.0084)	− 0.0051 (0.0091)	− 0.0078 (0.01)	− 0.005 (0.0082)	− 0.0057 (0.0099)	− 0.0103 (0.0131)	− 0.0067 (0.0105)	− 0.0077 (0.0125)
1	25	5000	− 0.0076 (0.0079)	− 0.0056 (0.006)	− 0.0053 (0.0077)	− 0.008 (0.0084)	− 0.0046 (0.0052)	− 0.0062 (0.0087)	− 0.01 (0.0105)	− 0.0058 (0.0066)	− 0.0081 (0.0103)
1	25	10,000	− 0.0077 (0.0079)	− 0.0056 (0.0058)	− 0.0063 (0.0078)	− 0.0081 (0.0083)	− 0.0046 (0.0049)	− 0.0067 (0.0085)	− 0.0101 (0.0103)	− 0.0058 (0.0063)	− 0.0087 (0.0104)
1	40	800	− 0.0131 (0.0149)	− 0.0111 (0.0132)	− 0.0113 (0.0142)	− 0.0161 (0.0184)	− 0.0137 (0.0163)	− 0.0139 (0.0176)	− 0.0186 (0.0219)	− 0.0152 (0.019)	− 0.0162 (0.0209)
1	40	5000	− 0.0169 (0.0177)	− 0.0149 (0.0159)	− 0.015 (0.0166)	− 0.0165 (0.0169)	− 0.0133 (0.0138)	− 0.0144 (0.0158)	− 0.0186 (0.0192)	− 0.0147 (0.0153)	− 0.0163 (0.0179)
1	40	10,000	− 0.0191 (0.0196)	− 0.0172 (0.0179)	− 0.0175 (0.0186)	− 0.0166 (0.0168)	− 0.0134 (0.0137)	− 0.0152 (0.0163)	− 0.0186 (0.0189)	− 0.0146 (0.0149)	− 0.0166 (0.0179)
1	60	800	− 0.0252 (0.0268)	− 0.0232 (0.0249)	− 0.0236 (0.0258)	− 0.0416 (0.0452)	− 0.0399 (0.0434)	− 0.0394 (0.0436)	− 0.0352 (0.0393)	− 0.0322 (0.0365)	− 0.0322 (0.0373)
1	60	5000	− 0.0256 (0.0259)	− 0.0231 (0.0235)	− 0.0244 (0.0253)	− 0.0468 (0.0474)	− 0.0449 (0.0455)	− 0.0431 (0.0444)	− 0.0346 (0.0353)	− 0.0309 (0.0316)	− 0.0317 (0.0331)
1	60	10,000	− 0.026 (0.0264)	− 0.0235 (0.024)	− 0.0249 (0.0258)	− 0.0469 (0.0473)	− 0.0449 (0.0452)	− 0.0426 (0.0434)	− 0.0349 (0.0352)	− 0.0312 (0.0315)	− 0.0315 (0.0326)
0.5	0.25	800	− 0.0012 (0.002)	− 0.0002 (0.002)	− 0.0004 (0.0056)	− 0.0047 (0.0061)	− 0.0036 (0.0054)	− 0.0042 (0.0075)	− 0.0083 (0.0107)	− 0.0071 (0.0099)	− 0.0074 (0.0114)
0.5	25	5000	− 0.0012 (0.0013)	0.0003 (0.0008)	− 0.0005 (0.005)	− 0.0048 (0.005)	− 0.0033 (0.0037)	− 0.0039 (0.0065)	− 0.0084 (0.0088)	− 0.0068 (0.0073)	− 0.0078 (0.0098)
0.5	25	10,000	− 0.0012 (0.0012)	0.0003 (0.0006)	− 0.0004 (0.005)	− 0.0047 (0.0049)	− 0.0032 (0.0034)	− 0.0044 (0.0069)	− 0.0084 (0.0086)	− 0.0067 (0.007)	− 0.0077 (0.0094)
0.5	40	800	− 0.0062 (0.0072)	− 0.0053 (0.0065)	− 0.0052 (0.0082)	− 0.0127 (0.0142)	− 0.0118 (0.0133)	− 0.0118 (0.0143)	− 0.0168 (0.0191)	− 0.0156 (0.0181)	− 0.0158 (0.0192)
0.5	40	5000	− 0.0063 (0.0064)	− 0.0051 (0.0053)	− 0.0057 (0.0076)	− 0.0129 (0.0131)	− 0.0116 (0.0119)	− 0.0122 (0.0136)	− 0.0167 (0.0172)	− 0.0153 (0.0157)	− 0.016 (0.0173)
0.5	40	10,000	− 0.0063 (0.0064)	− 0.0051 (0.0052)	− 0.0057 (0.0076)	− 0.0128 (0.0129)	− 0.0115 (0.0117)	− 0.012 (0.0132)	− 0.0167 (0.0169)	− 0.0152 (0.0155)	− 0.0162 (0.0172)
0.5	60	800	− 0.0669 (0.068)	− 0.0663 (0.0674)	− 0.0661 (0.0674)	− 0.0452 (0.0471)	− 0.0447 (0.0465)	− 0.0442 (0.0463)	− 0.0374 (0.0406)	− 0.0369 (0.0402)	− 0.0361 (0.0399)
0.5	60	5000	− 0.067 (0.0672)	− 0.0661 (0.0663)	− 0.0666 (0.067)	− 0.0451 (0.0454)	− 0.0441 (0.0444)	− 0.0447 (0.0454)	− 0.038 (0.0385)	− 0.0369 (0.0374)	− 0.0373 (0.0383)
0.5	60	10,000	− 0.0672 (0.0673)	− 0.0662 (0.0663)	− 0.0668 (0.067)	− 0.045 (0.0451)	− 0.0439 (0.0441)	− 0.0443 (0.0448)	− 0.0377 (0.0379)	− 0.0366 (0.0369)	− 0.0371 (0.0379)

The results of exploring the percentile of 80% of survival time in Table 3 reveal that the DBN model is superior in bias (RMSE) reduction in all the scenarios except one. When the data comes from survival distribution with shape parameter 0.5 and the censoring rate increases across time (alpha c = 2), for the sample sizes of 5000 with 60% censoring, the bias (RMSE) of Cox regression is − 0.1373 (0.1345). DBN's bias (RMSE) for this scenario is − 0.1375 (0.138). We should consider the increasing RMSE according to heavy censoring in this scenario. It causes more variation for Cox results, leading to the unstable mean of bias values.

**Table 3:** The models' bias and RMSE in survival probability estimation in percentile 80% of real survival time

*α*_S_	R(%)	N	*α*_C_ = 2			*α*_C_ = 1			*α*_C_ = 0.5		
			KM	Cox	DBN	KM	Cox	DBN	KM	Cox	DBN
2	25	800	− 0.0172 (0.0193)	− 0.0217 (0.0233)	− 0.0102 (0.015)	− 0.011 (0.0145)	− 0.0134 (0.016)	− 0.0038 (0.012)	− 0.008 (0.0123)	− 0.0087 (0.012)	− 0.0002 (0.011)
2	25	5000	− 0.017 (0.0174)	− 0.0209 (0.0212)	− 0.0107 (0.012)	− 0.011 (0.0115)	− 0.0127 (0.0131)	− 0.0037 (0.007)	− 0.0081 (0.0089)	− 0.0083 (0.0089)	− 0.0004 (0.006)
2	25	10,000	− 0.0171 (0.0173)	− 0.0209 (0.021)	− 0.0115 (0.0125)	− 0.0111 (0.0114)	− 0.0128 (0.013)	− 0.0053 (0.0071)	− 0.0082 (0.0086)	− 0.0082 (0.0085)	− 0.0028 (0.0056)
2	40	800	− 0.0353 (0.0382)	− 0.04 (0.0423)	− 0.0284 (0.0328)	− 0.0207 (0.0247)	− 0.0235 (0.0264)	− 0.013 (0.0199)	− 0.0141 (0.0189)	− 0.0154 (0.019)	− 0.006 (0.0157)
2	40	5000	− 0.0363 (0.0369)	− 0.0404 (0.0408)	− 0.0278 (0.0288)	− 0.0209 (0.0216)	− 0.0235 (0.024)	− 0.0116 (0.0135)	− 0.0139 (0.0148)	− 0.0145 (0.0152)	− 0.0046 (0.009)
2	40	10,000	− 0.0363 (0.0365)	− 0.0404 (0.0406)	− 0.0275 (0.0281)	− 0.0205 (0.0208)	− 0.0231 (0.0234)	− 0.0115 (0.0129)	− 0.0138 (0.0143)	− 0.0143 (0.0147)	− 0.0059 (0.0082)
2	60	800	− 0.0692 (0.0741)	− 0.0675 (0.0719)	− 0.0621 (0.068)	− 0.0408 (0.047)	− 0.0443 (0.0491)	− 0.0326 (0.0415)	− 0.0265 (0.0333)	− 0.0281 (0.033)	− 0.0185 (0.0284)
2	60	5000	− 0.0746 (0.0761)	− 0.0729 (0.0745)	− 0.0636 (0.0657)	− 0.0414 (0.0425)	− 0.0439 (0.0447)	− 0.0294 (0.0316)	− 0.0248 (0.0262)	− 0.0264 (0.0273)	− 0.0129 (0.0166)
2	60	10,000	− 0.0791 (0.0802)	− 0.0783 (0.0796)	− 0.0658 (0.0675)	− 0.0408 (0.0413)	− 0.0434 (0.0438)	− 0.0263 (0.0276)	− 0.0251 (0.0258)	− 0.0265 (0.027)	− 0.0115 (0.0139)
1	25	800	− 0.0224 (0.0241)	− 0.024 (0.0255)	− 0.0196 (0.0221)	− 0.0152 (0.0178)	− 0.016 (0.0183)	− 0.0122 (0.0161)	− 0.0099 (0.0132)	− 0.0099 (0.0132)	− 0.0074 (0.0127)
1	25	5000	− 0.0228 (0.023)	− 0.0237 (0.024)	− 0.0196 (0.0204)	− 0.0153 (0.0157)	− 0.0156 (0.016)	− 0.0115 (0.0129)	− 0.0097 (0.0104)	− 0.0092 (0.0098)	− 0.0057 (0.0082)
1	25	10,000	− 0.0227 (0.0228)	− 0.0236 (0.0237)	− 0.0201 (0.0206)	− 0.0153 (0.0155)	− 0.0155 (0.0157)	− 0.0122 (0.0132)	− 0.0098 (0.0101)	− 0.0092 (0.0095)	− 0.0066 (0.0084)
1	40	800	− 0.0374 (0.0401)	− 0.0376 (0.0403)	− 0.0346 (0.0379)	− 0.0311 (0.0345)	− 0.0325 (0.0356)	− 0.0282 (0.0324)	− 0.0189 (0.0232)	− 0.0197 (0.0234)	− 0.0161 (0.0216)
1	40	5000	− 0.0487 (0.0505)	− 0.0488 (0.0507)	− 0.0453 (0.0475)	− 0.0309 (0.0314)	− 0.0316 (0.032)	− 0.0271 (0.0282)	− 0.0181 (0.0189)	− 0.0183 (0.019)	− 0.014 (0.016)
1	40	10,000	− 0.0541 (0.0552)	− 0.0543 (0.0554)	− 0.0509 (0.0523)	− 0.0309 (0.0312)	− 0.0315 (0.0318)	− 0.0269 (0.0277)	− 0.0185 (0.0189)	− 0.0185 (0.0189)	− 0.0143 (0.0156)
1	60	800	− 0.0868 (0.0922)	− 0.086 (0.0915)	− 0.0839 (0.0895)	− 0.0637 (0.0692)	− 0.0641 (0.0693)	− 0.0604 (0.0666)	− 0.0354 (0.042)	− 0.0385 (0.044)	− 0.0321 (0.0396)
1	60	5000	− 0.1131 (0.1147)	− 0.1115 (0.113)	− 0.1101 (0.1119)	− 0.0702 (0.0714)	− 0.0708 (0.0719)	− 0.0661 (0.0676)	− 0.0352 (0.0364)	− 0.0367 (0.0378)	− 0.0307 (0.0327)
1	60	10,000	− 0.1175 (0.1179)	− 0.1157 (0.1161)	− 0.1142 (0.1149)	− 0.0705 (0.071)	− 0.071 (0.0715)	− 0.066 (0.0669)	− 0.035 (0.0356)	− 0.0363 (0.0368)	− 0.0303 (0.0316)
0.5	0.25	800	− 0.0295 (0.0305)	− 0.0304 (0.0314)	− 0.0288 (0.0302)	− 0.0228 (0.0243)	− 0.0238 (0.0252)	− 0.0217 (0.0238)	− 0.0148 (0.0173)	− 0.0156 (0.018)	− 0.0139 (0.0174)
0.5	25	5000	− 0.0301 (0.0302)	− 0.0305 (0.0306)	− 0.029 (0.0295)	− 0.022 (0.0223)	− 0.0224 (0.0227)	− 0.021 (0.0218)	− 0.0146 (0.015)	− 0.0148 (0.0152)	− 0.0136 (0.0147)
0.5	25	10,000	− 0.0302 (0.0303)	− 0.0305 (0.0306)	− 0.0288 (0.0293)	− 0.0219 (0.022)	− 0.0222 (0.0224)	− 0.0205 (0.0211)	− 0.0149 (0.0151)	− 0.015 (0.0153)	− 0.0137 (0.0147)
0.5	40	800	− 0.1071 (0.1089)	− 0.108 (0.1098)	− 0.105 (0.107)	− 0.0567 (0.0592)	− 0.0579 (0.0603)	− 0.0557 (0.0584)	− 0.029 (0.0324)	− 0.0303 (0.0334)	− 0.0275 (0.0315)
0.5	40	5000	− 0.1079 (0.1082)	− 0.1079 (0.1082)	− 0.1072 (0.1076)	− 0.0565 (0.0569)	− 0.0569 (0.0573)	− 0.0554 (0.0561)	− 0.0294 (0.03)	− 0.03 (0.0305)	− 0.0283 (0.0294)
0.5	40	10,000	− 0.1075 (0.1077)	− 0.1075 (0.1077)	− 0.1067 (0.107)	− 0.0564 (0.0566)	− 0.0567 (0.0569)	− 0.0554 (0.0558)	− 0.0292 (0.0295)	− 0.0297 (0.03)	− 0.0279 (0.0286)
0.5	60	800	− 0.131 (0.1359)	− 0.1318 (0.1345)	− 0.128 (0.1324)	− 0.0951 (0.1221)	− 0.113 (0.1276)	− 0.0869 (0.1134)	− 0.0673 (0.0734)	− 0.0704 (0.076)	− 0.0653 (0.0718)
0.5	60	5000	− 0.1382 (0.1385)	− 0.1373 (0.1377)	− 0.1375 (0.138)	− 0.1655 (0.1679)	− 0.166 (0.1683)	− 0.1619 (0.1653)	− 0.0673 (0.0683)	− 0.0688 (0.0697)	− 0.0665 (0.0677)
0.5	60	10,000	− 0.1305 (0.1398)	− 0.1329 (0.1359)	− 0.1302 (0.1381)	− 0.1637 (0.1649)	− 0.1638 (0.1649)	− 0.162 (0.1633)	− 0.067 (0.0675)	− 0.0683 (0.0688)	− 0.0663 (0.0671)

### Real data analysis

The baseline characteristics of patients by their status in the last observation are summarized in Table 4. The pathology exam for the 672 (88%) patients resulted in adenocarcinoma; however, the prevalence did not differ significantly across groups. The total gastrectomy was the most prevalent procedure, with 403 (53%). However, the death event was distributed almost equally in all the surgery types (*P*-value = 0.3). As a predictable pattern, the metastasis cases were less frequent in survivors (17% of them survived compared to 83% of death cases). In addition, the survivors were almost categorized into the lower Stages.

**Table 4:** Descriptive statistics of patients by the last observation status

Variable	Overall, N = 760	Death, N = 573	Survivor, N = 187	*P*-value^a^
	n (%)	n (%)	n (%)	
Pathology				0.053
Adeno	672 (88)	514 (77)	158 (24)	
Other	88 (12)	59 (67)	29 (33)	
Surgery				0.257
Total gastrectomy	403 (53)	306 (76)	97 (24)	
Subtotal gastrectomy	194 (26)	142 (73)	52 (27)	
Distal gastrectomy	32 (4.2)	20 (63)	12 (38)	
Partial gastrectomy	59 (7.8)	46 (78)	13 (22)	
Proximal gastrectomy	72 (9.5)	59 (82)	13 (18)	
Age				< 0.001
< 61	184 (24)	106 (58)	78 (42)	
61–70	363 (48)	266 (73)	97 (27)	
> 70	213 (28)	201 (94)	12 (6)	
Sex				0.957
Female	241 (32)	182 (75)	59 (25)	
Male	519 (68)	391 (75)	128 (25)	
Smoking				0.759
Non-smoker	523 (69)	396 (76)	127 (24)	
Smoker	237 (31)	177 (75)	60 (25)	
Site				0.102
Cardia	341 (45)	263 (77)	78 (23)	
Antrum	150 (20)	103 (69)	47 (31)	
Other	269 (35)	207 (77)	62 (23)	
Metastasis				< 0.001
Non-Metastasis	321 (42)	208 (65)	113 (35)	
Metastasis	439 (58)	365 (83)	74 (17)	
Stage				< 0.001
I	61 (8.0)	31 (51)	30 (49)	
II	265 (35)	182 (69)	83 (31)	
III	295 (39)	237 (80)	58 (20)	
IV	139 (18)	123 (89)	16 (12)	

^a^Pearson's Chi-squared test

**Fig. 2:**
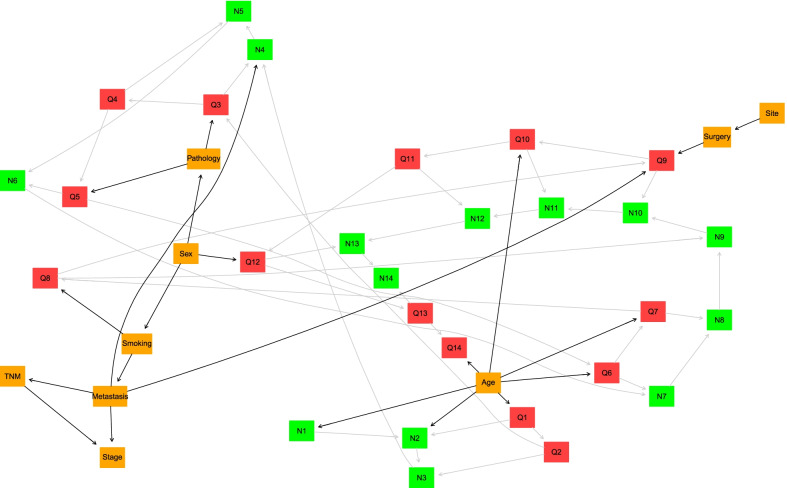
Representation of DAG corresponds to survival analysis of real-world data (survival of patients after gastrectomy) surgery

**Fig. 3:**
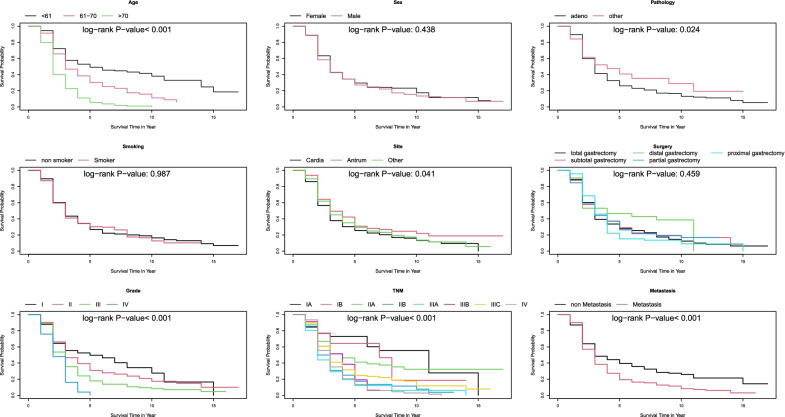
Kaplan–Meier survival probability curves of patients according to their baseline characteristics and the results of the log-rank test to compare the curves

**Fig. 4:**
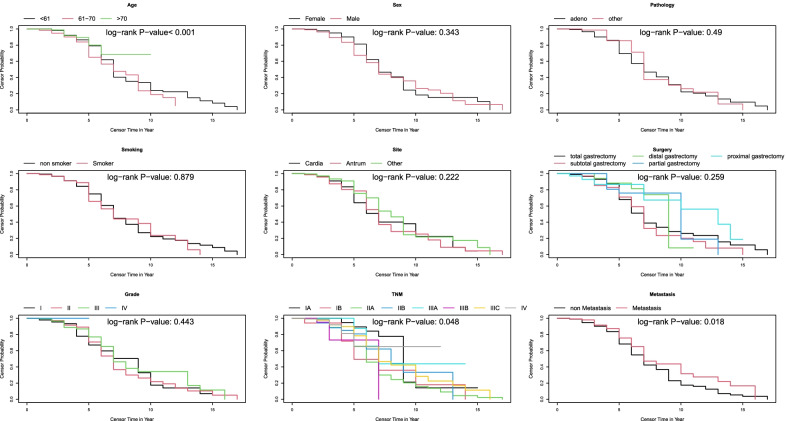
The censorship probability curve of patients according to their baseline characteristics and the results of the log-rank test to compare the curves

We used the Tabu search algorithm based on the BDE score function, considering the clinical justifications and validation results. The final DAG is presented in Fig. 2. For a better exploration, the survival and censor probability curves are depicted in Figs. 3 and 4. According to the DAG in Fig. 2, the baseline age is related to survival in the first- and second years post-operative. The corresponding Kaplan–Meier curve in Fig. 3 confirmed this finding, and the survival lines diverge in the initial years and continue to be parallel. On the other hand, there is an edge between metastatic status and $N_{4}$ in DAG. The survival lines in Fig. 3 for metastasis and non-metastasis cases started to get away from each other at this time point.

On the other hand, the directed edges from age to $Q_{6}$ and $Q_{7}$ in DAG correspond to flattening the censor probability after year 6 for more than 70 years old patients and a sharp decrease in censor probability less than 61 in year seven that leads to crossing another line (Fig. 4). The censor probability lines of adenocarcinoma and the other group separate from each other at years 3 and 5 in Fig. 4. It is coordinated to edges from the pathology node to $Q_{3}$ and $Q_{5}$. All the other edges from the covariates to $Q_{t}$ nodes in DAG correspond to a specific pattern in Fig. 4 and are justifiable.

According to Table 5, the higher baseline age is the most critical factor for experiencing the death event early. The hazards for 61–70 and more than 70 years old patients are 1.77 (95% CI 1.40–2.24) and 3.99 (95% CI 3.09–5.14) times for those less than 61 years old. The edges from the age node to $N_{1}$ and $N_{2}$ in Fig. 2 assert that the effect of age is more notable in the initial times.

**Table 5:** The results of the univariable and multivariable Cox PH model

Characteristic (Reference)	Univariable			Multivariable		
	HR^a^	95% CI^a^	*P*-value	HR^a^	95% CI^a^	*P*-value
Pathology (Other)						
Adeno	1.37	1.04, 1.79	**0.023**	1.33	1.00, 1.78	**0.048**
Surgery (Total gastrectomy)						
Subtotal gastrectomy	0.96	0.79, 1.17	0.683	1.01	0.81, 1.25	0.941
Distal gastrectomy	0.67	0.42, 1.05	0.080	1.07	0.65, 1.75	0.799
Partial gastrectomy	0.95	0.70, 1.30	0.758	1.02	0.74, 1.41	0.911
Proximal gastrectomy	1.04	0.78, 1.37	0.808	0.86	0.64, 1.15	0.302
Age (< 61)						
61–70	1.74	1.38, 2.19	**< 0.001**	1.77	1.40, 2.24	**< 0.001**
> 70	3.73	2.91, 4.77	**< 0.001**	3.99	3.09, 5.14	**< 0.001**
Sex (Female)						
Male	1.07	0.90, 1.28	0.438	1.04	0.86, 1.27	0.680
Smoking (Non-smoker)						
Smoker	1.00	0.84, 1.19	0.975	0.95	0.78, 1.15	0.595
Site (Other)						
Antrum	0.86	0.68, 1.09	0.179	0.81	0.64, 1.04	0.102
Cardia	1.13	0.94, 1.36	0.207	1.17	0.96, 1.43	0.128
Metastasis (Non-Metastasis)						
Metastasis	1.52	1.28, 1.81	**< 0.001**	3.89	1.57, 9.62	**0.003**
Stage (I)						
II	1.96	1.33, 2.87	**< 0.001**	1.95	1.29, 2.94	**0.002**
III	2.38	1.63, 3.46	**< 0.001**	0.54	0.20, 1.45	0.219
IV	3.00	2.01, 4.46	**< 0.001**	0.85	0.31, 2.32	0.755

^a^Pearson's Chi-squared test

The stage and metastasis reflect two relatively same aspects of disease progression in surgery time. Therefore, there is some degree of correlation between these variables. That is why the stage was no longer significant in the multivariable model when we added metastasis. The metastasis cases had a 3.89 (95% CI 1.57–9.62) times higher hazard than the others. In contrast to the DBN, the Cox model does not ensure us about the relations between these variables. We present the conditional probability tables of the model covariates in the Additional file 1 for more clarification.

As the results of model validation, the mean posterior classification errors and their standard deviation for the whole learned network and $N_{t}$, and $Q_{t}$ nodes are represented in the Additional file 1. The expected loss for all the scenarios did not exceed the acceptable value of 0.04, which means all the networks predicted the state variables with more than 96% accuracy.

## Discussion

We extended the classical idea of the Kaplan–Meier estimator and used the BN facilities to make a novel model for analyzing the survival data. The Bayesian network tools enable us to explore the different aspects of data in a previously impossible way. For instance, nonparametric survival methods like Kaplan–Meier were not adjusted to take covariates into account. On the other hand, the regression approaches only focus on the outcome variable and ignore the relations between covariates. The majority of the survival models were developed according to strict assumptions. In most applied cases, checking these assumptions is ignored or even hard to satisfy. Our model addressed the issues of the previous approaches and required the least possible assumptions.

Censorship which leads to incomplete observations, is an intrinsic property of survival data. Methods developed in this domain tried to manage this issue and incorporate the information of the censored observation as much as possible. Many researchers in the setting of machine learning ignore the censor observation and change the problem to explore the continuous-time outcome variable [5, 17, 28, 29]. In contrast, we consider a state for censorship that enables the model to examine how covariates affect this state. This property significantly increases the prediction power of the model. In real-world applications, administrators of data registries could manage situations to avoid preventable censorship.

The graphical aspect and conditional probability distributions of BN reflect much information in the simplest form. In comparison, other survival base algorithms in machine learning, like neural networks [18–21], support vector machines [22, 23], and ensemble models [24, 25], are as much sophisticated in outlining the patterns of data. On the other hand, alternative classical approaches like the Cox PH, frailty concept, and the other parametric regression models [1] involves the users in the intricate interpretations of their effect sizes.

The DAG of the BN model is the only mechanism for demonstrating the intra-relationship of covariates which is not available in the alternative approaches. For instance, interventions could be designed basis on the roots of the network or the parents of unchangeable nodes. In addition, this model feedback the correlation between variables, as we have seen in our example. The causal inference is one of the extensions of BNs, which we do not explore here [39]. Finally, the DBN forced the covariates to be discretized.

Using the semiparametric and parametric survival models should be cautionary. The Cox model's proportional hazard assumption violation leads to incorrect inferences or underestimating the hazard ratio [13, 40]. On the other hand, the parametric approach relies on the outcome distribution. These models assume a parametric distribution for the survival time, which is hard to satisfy, significantly when we have heavily censored data [41]. Finally, even the nonparametric approaches assume non-informative censorship [42]. Our BN has two primary assumptions. At first, the variables follow the Markov property, and then conditional multinomial or conditional binomial distribution is appropriate for discrete nodes. Both of these assumptions are logical in practice [43].

We conducted a wide range of scenarios in the simulation study. The DBN was superior to Kaplan–Meier in bias (RMSE) reduction in almost all of them. In addition, our results showed the comparability of Cox regression and DBN in this context. Our model was significantly superior to the Cox regression when the interest was exploring late survival times.

The Kaplan Meier biases were negative in all the scenarios. Hence, this method always estimates the survival probability as less than the actual value. Other simulation studies explain this issue [44, 45], and several suggest correction approaches. Stute and Wang proposed a Jackknife method to reduce the Kaplan Meier bias [46]. In another attempt, Jiang used the geometric mean of survival and censoring curves for bias correction [47].

In most scenarios that explore the lowest percentile with the lower sensor rate, the bias of Cox regression was estimated to be positive. In addition, the Cox biases were positive in the decreasing event rate and increasing censor rate for all the scenarios. Langner et al. showed that the maximum likelihood estimations of Cox are biased. They conducted a simulation study and concluded that there is a direct relationship between higher levels of event risk and seeing positive bias [48]. In concordance with their findings, we find that everywhere we expected to see the event, more than censoring the biases tend to the positive values.

The relation of covariates in our model is reasonably justifiable in the clinical aspect. Gender is the most known indicator of smoking across the population. According to the national representative survey, the age-standardized prevalence of current tobacco smoking among Iranian adults was 24.4% (95% CI 23.6–25.1) in males and 3.8% (95% CI 3.5–4.1) in females [49]. Therefore, it seems evident that the sex node is the parent of smoking.

We used the 7th version of the TNM Classification of Malignant Tumors (TNM) staging system for gastric cancer. The M parameter in TNM, representing distance metastases, is a critical prognostic for survival probability [50]. On the other hand, some studies described that the 7th TNM did not appropriately classify the biological behavior of cancer and the prognosis of patients [51]. In this manner, the 8th edition of the TNM staging with reforms to show relevant differences in stage III disease survival rates was released [52]. These arguments support our finding that TNM and stage nodes are affected by metastasis but are not the parents of any survival mechanism nodes.

Several studies on the Iranian population confirmed that a higher baseline age increases the hazard of death events in gastric patients who have undergone surgery. Interestingly, these studies did not mention a significant difference between males and females [53, 54].

## Conclusion

Our proposed DBN could be used as a data mining technique in the context of survival data analysis. The feature selection ability of this model is comparable with the Cox PH model in both statistical and clinical aspects. In contrast to the Kaplan–Meier, our model can handle high-dimensional data and does not require the restrictive assumptions of regression approaches. The available machine learning algorithms are relatively sophisticated and rarely consider the censorship property of survival data. Whereas BN is a straightforward method, the DBN incorporates the information of censoring observations in inferences.

In this study, we introduced the simplest DBN model for survival analysis and compared its performance to the most used methods in the clinical field. This model could be adjusted for a specific situation like competing risk, time-variant covariates, and high dimensional data. In this manner, more specific simulation studies would be required.

## Supplementary Information

**Additional file 1: Supplementary file 1.** The validation of structure learning. The posterior classification error of HC and Tabu algorithms for all nodes according to different score functions. **Supplementary file 2.** Conditional probability distribution of time stationary variables in the model. **Supplementary figure 1.** Conditional probability distribution of node stage given the different levels of its parents (Metastasis and TNM). **Supplementary figure 2.** Conditional probability distribution of node TNM given the different levels of its parent (Metastasis). **Supplementary figure 3.** Conditional probability distribution of node metastasis given the different levels of its parent (Smoking). **Supplementary figure 4.** Conditional probability distribution of node pathology given the different levels of its parent (Sex). **Supplementary figure 5.** Conditional probability distribution of node smoking given the different levels of its parent (Sex). **Supplementary figure 6.** Conditional probability distribution of node surgery given the different levels of its parent (Site).

## Data Availability

The datasets used and/or analysed during the current study available from the corresponding author on reasonable request.

## Abbreviations

TNM: TNM Classification of Malignant Tumors
DAG: Directed Acyclic Graph
BN: Bayesian Network
DBN: Dynamic Bayesian Network
2-TBN: 2-Slice Temporal Bayesian Network
PH: Proportional Hazard
HC: Hill-Climbing
