# Supplementary material for: A novel dynamic Bayesian network approach for data mining and survival data analysis
Source: BMC Med Inform Decis Mak. 2022 Sep 22;22:251. doi: 10.1186/s12911-022-02000-7 (PMC9503243; doi:10.1186/s12911-022-02000-7)
Supplement: Supplementary file 1 — Additional file 1: Supplementary file 1. The validation of structure learning. The posterior classification error of HC and Tabu algorithms for all nodes according to different score functions. Supplementary file 2. Conditional probability distribution of time stationary variables in the model. Supplementary figure 1. Conditional probability distribution of node stage given the different levels of its parents (Metastasis and TNM). Supplementary figure 2. Conditional probability distribution of node TNM given the different levels of its parent (Metastasis). Supplementary figure 3. Conditional probability distribution of node metastasis given the different levels of its parent (Smoking). Supplementary figure 4. Conditional probability distribution of node pathology given the different levels of its parent (Sex). Supplementary figure 5. Conditional probability distribution of node smoking given the different levels of its parent (Sex). Supplementary figure 6. Conditional probability distribution of node surgery given the different levels of its parent (Site). [file 12911_2022_2000_MOESM1_ESM.docx]

Supplementary file 1. The validation of structure learning. The posterior classification error of HC and Tabu algorithms for all nodes according to different score functions.

|  | HC | | | | | | Tabu | | | | | |
| --- | --- | --- | --- | --- | --- | --- | --- | --- | --- | --- | --- | --- |
| State | AIC | BDE | BDS | BIC | K2 | loglikelihood | AIC | BDE | BDS | BIC | K2 | loglikelihood |
| **N1** | 0.115 (0.017) | 0.120 (0.017) | 0.115 (0.017) | 0.121 (0.023) | 0.106 (0.020) | 0.102 (0.021) | 0.118 (0.019) | 0.112 (0.015) | 0.120 (0.030) | 0.109 (0.018) | 0.117 (0.020) | 0.106 (0.017) |
| **N2** | 0.183 (0.033) | 0.167 (0.021) | 0.180 (0.015) | 0.167 (0.023) | 0.187 (0.021) | 0.193 (0.052) | 0.183 (0.027) | 0.185 (0.022) | 0.191 (0.015) | 0.154 (0.017) | 0.167 (0.032) | 0.184 (0.025) |
| **N3** | 0.075 (0.019) | 0.082 (0.017) | 0.080 (0.011) | 0.076 (0.012) | 0.074 (0.016) | 0.092 (0.020) | 0.085 (0.027) | 0.080 (0.014) | 0.076 (0.020) | 0.077 (0.011) | 0.071 (0.019) | 0.078 (0.018) |
| **N4** | 0.071 (0.017) | 0.056 (0.015) | 0.062 (0.021) | 0.060 (0.015) | 0.054 (0.010) | 0.028 (0.014) | 0.061 (0.014) | 0.067 (0.016) | 0.064 (0.024) | 0.054 (0.013) | 0.056 (0.014) | 0.027 (0.020) |
| **N5** | 0.025 (0.012) | 0.029 (0.011) | 0.028 (0.010) | 0.019 (0.011) | 0.025 (0.007) | 0.018 (0.014) | 0.027 (0.012) | 0.027 (0.012) | 0.033 (0.013) | 0.018 (0.008) | 0.025 (0.011) | 0.011 (0.010) |
| **N6** | 0.008 (0.004) | 0.011 (0.004) | 0.011 (0.007) | 0.010 (0.004) | 0.012 (0.008) | 0.000 (0.000) | 0.009 (0.005) | 0.012 (0.006) | 0.006 (0.002) | 0.009 (0.004) | 0.009 (0.008) | 0.000 (0.000) |
| **N7** | 0.010 (0.006) | 0.010 (0.008) | 0.010 (0.008) | 0.009 (0.006) | 0.010 (0.004) | 0.013 (0.011) | 0.012 (0.006) | 0.012 (0.004) | 0.012 (0.005) | 0.011 (0.007) | 0.012 (0.004) | 0.008 (0.010) |
| **N8** | 0.0034 (0.0032) | 0.0068 (0.0048) | 0.0041 (0.0026) | 0.0058 (0.0030) | 0.0023 (0.0045) | 0.0000 (0.0000) | 0.0033 (0.0032) | 0.0055 (0.0036) | 0.0055 (0.0048) | 0.0053 (0.0068) | 0.0050 (0.0045) | 0.0000 (0.0000) |
| **N9** | 0.007 (0.005) | 0.006 (0.005) | 0.004 (0.004) | 0.004 (0.004) | 0.007 (0.004) | 0.000 (0.000) | 0.0066 (0.0052) | 0.0037 (0.0047) | 0.0032 (0.0031) | 0.0026 (0.0031) | 0.0047 (0.0033) | 0.0000 (0.0000) |
| **N10** | 0.008 (0.005) | 0.005 (0.003) | 0.011 (0.007) | 0.008 (0.007) | 0.007 (0.007) | 0.002 (0.008) | 0.005 (0.006) | 0.005 (0.005) | 0.005 (0.005) | 0.006 (0.005) | 0.008 (0.007) | 0.001 (0.005) |
| **N11** | 0.0042 (0.0041) | 0.0027 (0.0038) | 0.0018 (0.0032) | 0.0027 (0.0031) | 0.0023 (0.0038) | 0.0000 (0.0000) | 0.0024 (0.0025) | 0.0014 (0.0022) | 0.0032 (0.0038) | 0.0013 (0.0030) | 0.0030 (0.0043) | 0.0000 (0.0000) |
| **N12** | 0.0014 (0.0023) | 0.0032 (0.0031) | 0.0023 (0.0024) | 0.0027 (0.0038) | 0.0027 (0.0038) | 0.0000 (0.0000) | 0.0010 (0.0020) | 0.0014 (0.0022) | 0.0019 (0.0040) | 0.0013 (0.0021) | 0.0019 (0.0024) | 0.0000 (0.0000) |
| **N13** | 0.0037 (0.0029) | 0.0019 (0.0024) | 0.0041 (0.0026) | 0.0027 (0.0023) | 0.0018 (0.0023) | 0.0000 (0.0000) | 0.0023 (0.0025) | 0.0023 (0.0024) | 0.0027 (0.0023) | 0.0035 (0.0028) | 0.0024 (0.0025) | 0.0000 (0.0000) |
| **N14** | 0.0019 (0.0024) | 0.0023 (0.0024) | 0.0027 (0.0038) | 0.0022 (0.0023) | 0.0023 (0.0024) | 0.0000 (0.0000) | 0.0010 (0.0020) | 0.0005 (0.0014) | 0.0014 (0.0022) | 0.0022 (0.0023) | 0.0018 (0.0023) | 0.0000 (0.0000) |
| **Q1** | 0.114 (0.017) | 0.115 (0.017) | 0.113 (0.023) | 0.116 (0.023) | 0.112 (0.014) | 0.124 (0.032) | 0.119 (0.020) | 0.116 (0.021) | 0.115 (0.013) | 0.115 (0.019) | 0.108 (0.022) | 0.104 (0.018) |
| **Q2** | 0.191 (0.020) | 0.199 (0.047) | 0.184 (0.016) | 0.174 (0.015) | 0.179 (0.023) | 0.174 (0.031) | 0.186 (0.018) | 0.180 (0.025) | 0.199 (0.031) | 0.164 (0.019) | 0.182 (0.027) | 0.173 (0.032) |
| **Q3** | 0.081 (0.017) | 0.092 (0.021) | 0.083 (0.016) | 0.064 (0.013) | 0.076 (0.018) | 0.073 (0.026) | 0.089 (0.020) | 0.094 (0.027) | 0.086 (0.016) | 0.072 (0.021) | 0.092 (0.013) | 0.088 (0.015) |
| **Q4** | 0.064 (0.009) | 0.060 (0.015) | 0.064 (0.016) | 0.051 (0.013) | 0.049 (0.014) | 0.025 (0.012) | 0.066 (0.015) | 0.059 (0.013) | 0.066 (0.015) | 0.064 (0.019) | 0.063 (0.012) | 0.019 (0.006) |
| **Q5** | 0.023 (0.009) | 0.028 (0.011) | 0.025 (0.007) | 0.024 (0.008) | 0.022 (0.008) | 0.018 (0.012) | 0.027 (0.011) | 0.030 (0.007) | 0.021 (0.010) | 0.024 (0.008) | 0.016 (0.006) | 0.016 (0.008) |
| **Q6** | 0.015 (0.006) | 0.008 (0.004) | 0.008 (0.003) | 0.010 (0.004) | 0.011 (0.007) | 0.000 (0.000) | 0.012 (0.006) | 0.017 (0.014) | 0.013 (0.006) | 0.009 (0.007) | 0.010 (0.006) | 0.000 (0.000) |
| **Q7** | 0.011 (0.004) | 0.010 (0.006) | 0.010 (0.006) | 0.012 (0.008) | 0.009 (0.007) | 0.009 (0.011) | 0.013 (0.008) | 0.010 (0.003) | 0.014 (0.010) | 0.012 (0.005) | 0.011 (0.008) | 0.005 (0.006) |
| **Q8** | 0.0047 (0.0044) | 0.0032 (0.0022) | 0.0054 (0.0046) | 0.0049 (0.0039) | 0.0040 (0.0040) | 0.0000 (0.0000) | 0.006 (0.004) | 0.009 (0.003) | 0.007 (0.005) | 0.004 (0.002) | 0.004 (0.003) | 0.000 (0.000) |
| **Q9** | 0.007 (0.007) | 0.004 (0.004) | 0.005 (0.004) | 0.004 (0.004) | 0.008 (0.005) | 0.000 (0.000) | 0.006 (0.007) | 0.006 (0.006) | 0.006 (0.007) | 0.007 (0.004) | 0.005 (0.005) | 0.000 (0.000) |
| **Q10** | 0.004 (0.002) | 0.006 (0.003) | 0.006 (0.004) | 0.008 (0.005) | 0.007 (0.004) | 0.000 (0.000) | 0.008 (0.006) | 0.007 (0.007) | 0.006 (0.006) | 0.008 (0.006) | 0.006 (0.005) | 0.001 (0.004) |
| **Q11** | 0.0042 (0.0042) | 0.0022 (0.0032) | 0.0018 (0.0023) | 0.0022 (0.0031) | 0.0023 (0.0044) | 0.0000 (0.0000) | 0.0014 (0.0023) | 0.0055 (0.0036) | 0.0036 (0.0036) | 0.0013 (0.0030) | 0.0033 (0.0031) | 0.0000 (0.0000) |
| **Q12** | 0.0020 (0.0034) | 0.0014 (0.0022) | 0.0005 (0.0015) | 0.0031 (0.0036) | 0.0013 (0.0030) | 0.0000 (0.0000) | 0.0009 (0.0029) | 0.0027 (0.0038) | 0.0013 (0.0030) | 0.0009 (0.0019) | 0.0019 (0.0024) | 0.0000 (0.0000) |
| **Q13** | 0.0028 (0.0024) | 0.0032 (0.0022) | 0.0041 (0.0026) | 0.0026 (0.0023) | 0.0038 (0.0020) | 0.0000 (0.0000) | 0.0038 (0.0037) | 0.0022 (0.0032) | 0.0045 (0.0021) | 0.0035 (0.0019) | 0.0023 (0.0025) | 0.0000 (0.0000) |
| **Q14** | 0.0015 (0.0023) | 0.0014 (0.0022) | 0.0028 (0.0024) | 0.0018 (0.0031) | 0.0047 (0.0032) | 0.0000 (0.0000) | 0.0009 (0.0020) | 0.0014 (0.0031) | 0.0009 (0.0019) | 0.0022 (0.0023) | 0.0015 (0.0025) | 0.0000 (0.0000) |
| **Total N** | 0.0368 (0.0020) | 0.0359 (0.0020) | 0.0368 (0.0035) | 0.0350 (0.0024) | 0.0352 (0.0024) | 0.0321 (0.0030) | 0.037 (0.004) | 0.037 (0.003) | 0.038 (0.005) | 0.032 (0.003) | 0.034 (0.003) | 0.030 (0.002) |
| **Total Q** | 0.038 (0.002) | 0.038 (0.004) | 0.037 (0.003) | 0.034 (0.003) | 0.035 (0.003) | 0.030 (0.005) | 0.039 (0.003) | 0.038 (0.003) | 0.039 (0.004) | 0.035 (0.003) | 0.036 (0.002) | 0.029 (0.003) |
| **Total** | 0.0372 (0.0015) | 0.0370 (0.0024) | 0.0367 (0.0024) | 0.0346 (0.0024) | 0.0351 (0.0023) | 0.0312 (0.0017) | 0.038 (0.003) | 0.038 (0.002) | 0.038 (0.004) | 0.034 (0.002) | 0.035 (0.002) | 0.029 (0.002) |
| ^1^Mean (SD) | | | | | | | | | | | | |

Supplementary file 2. Conditional probability distribution of time stationary variables in the model.


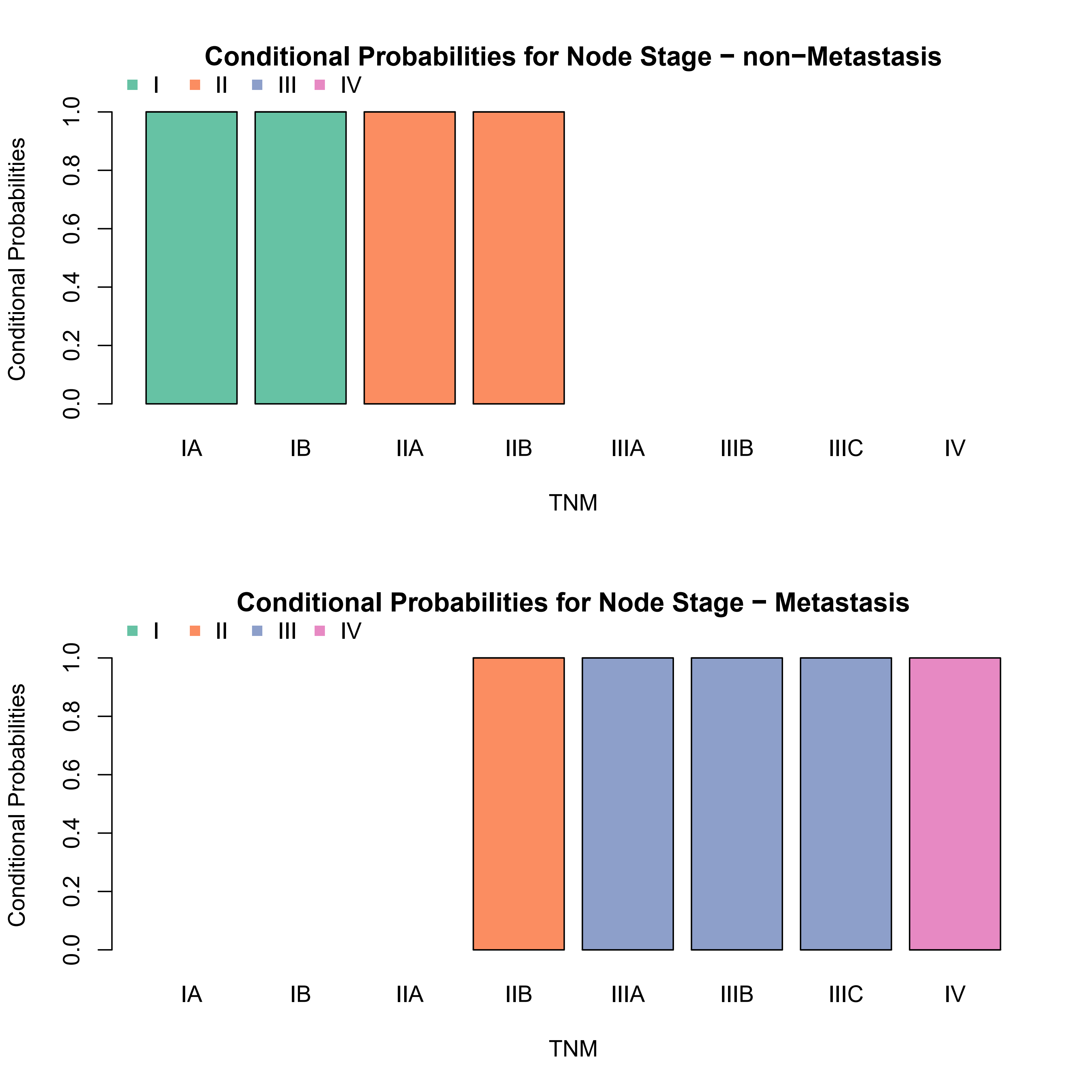


Supplementary figure 1. Conditional probability distribution of node stage given the different levels of its parents (Metastasis and TNM)


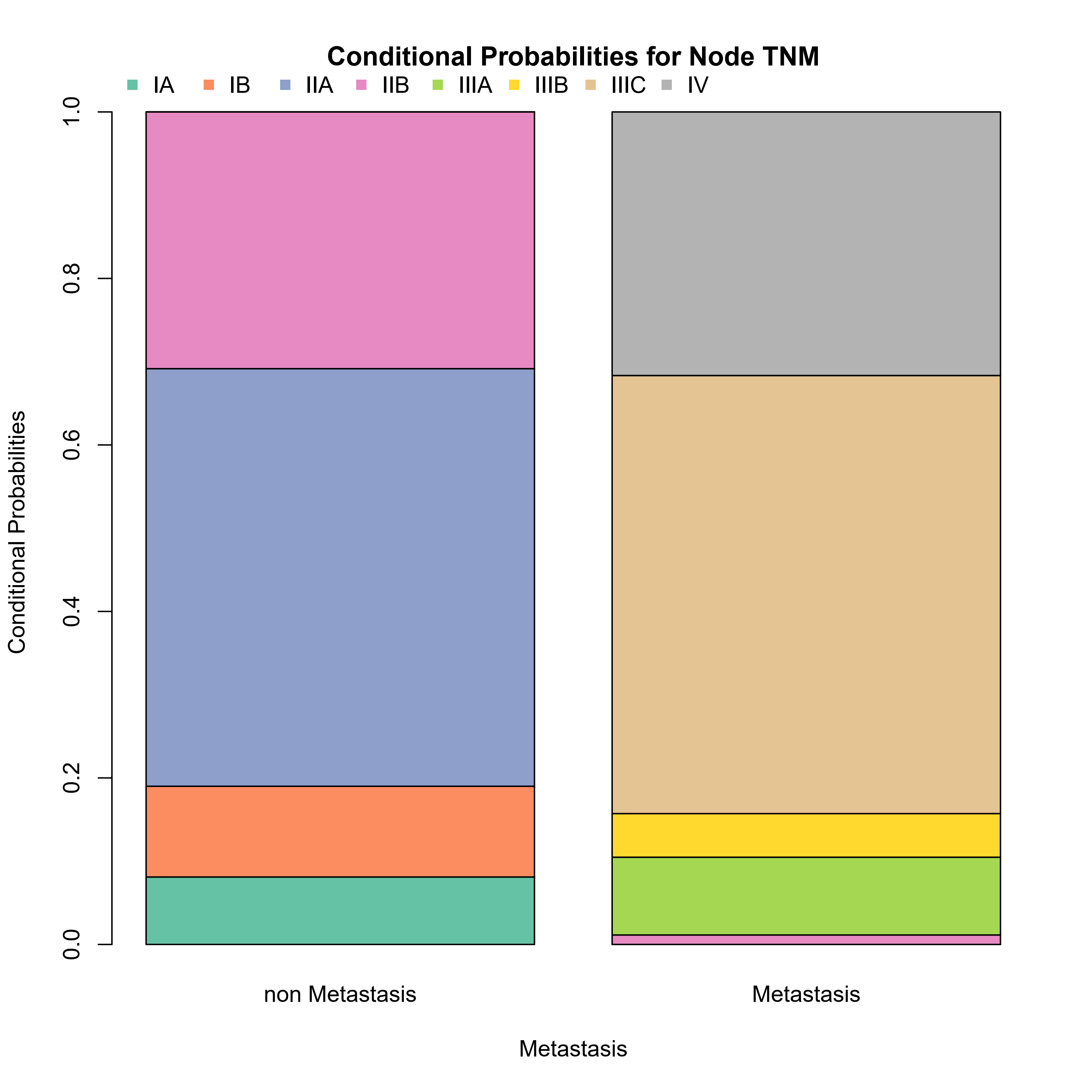


Supplementary figure 2. Conditional probability distribution of node TNM given the different levels of its parent (Metastasis)


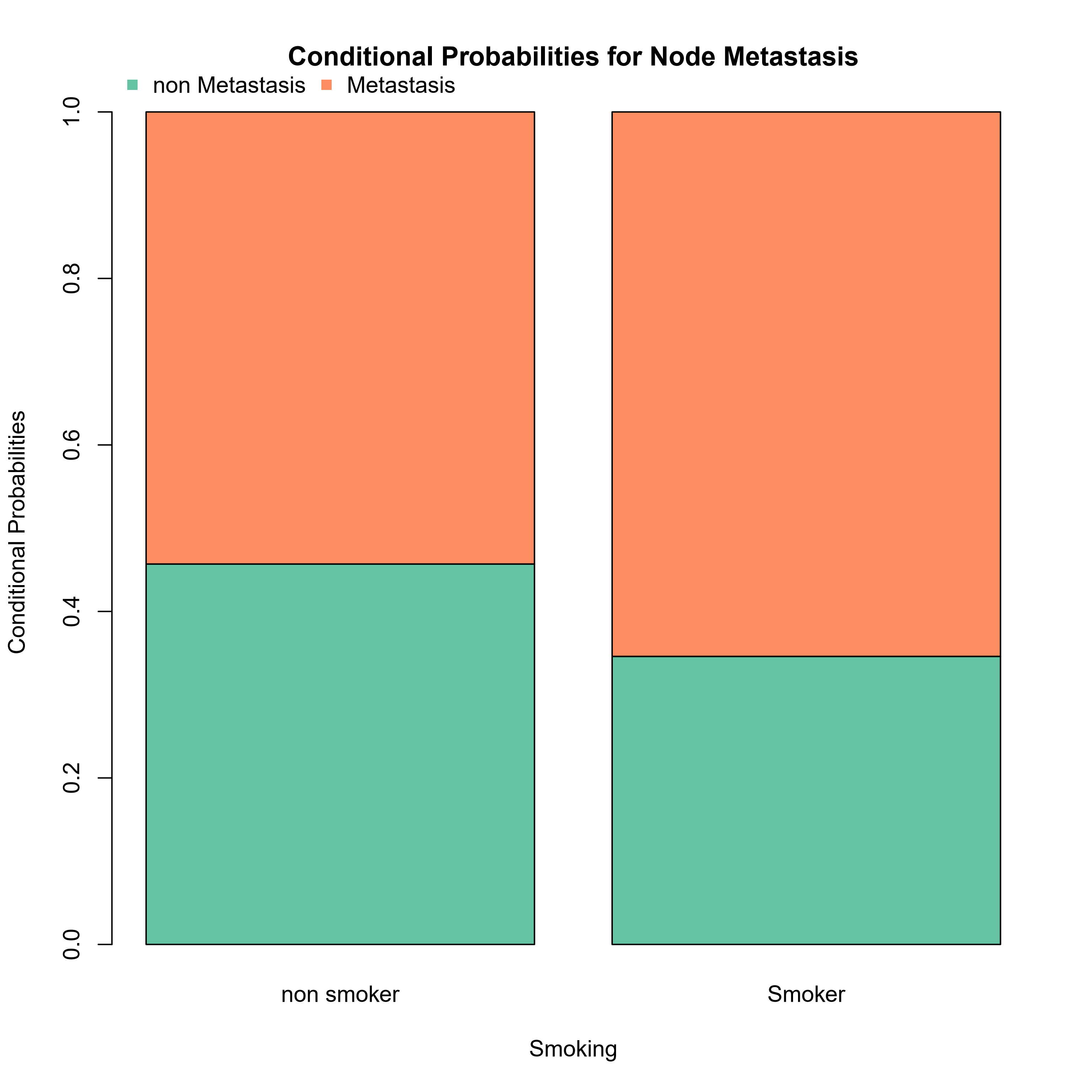


Supplementary figure 3. Conditional probability distribution of node metastasis given the different levels of its parent (Smoking)


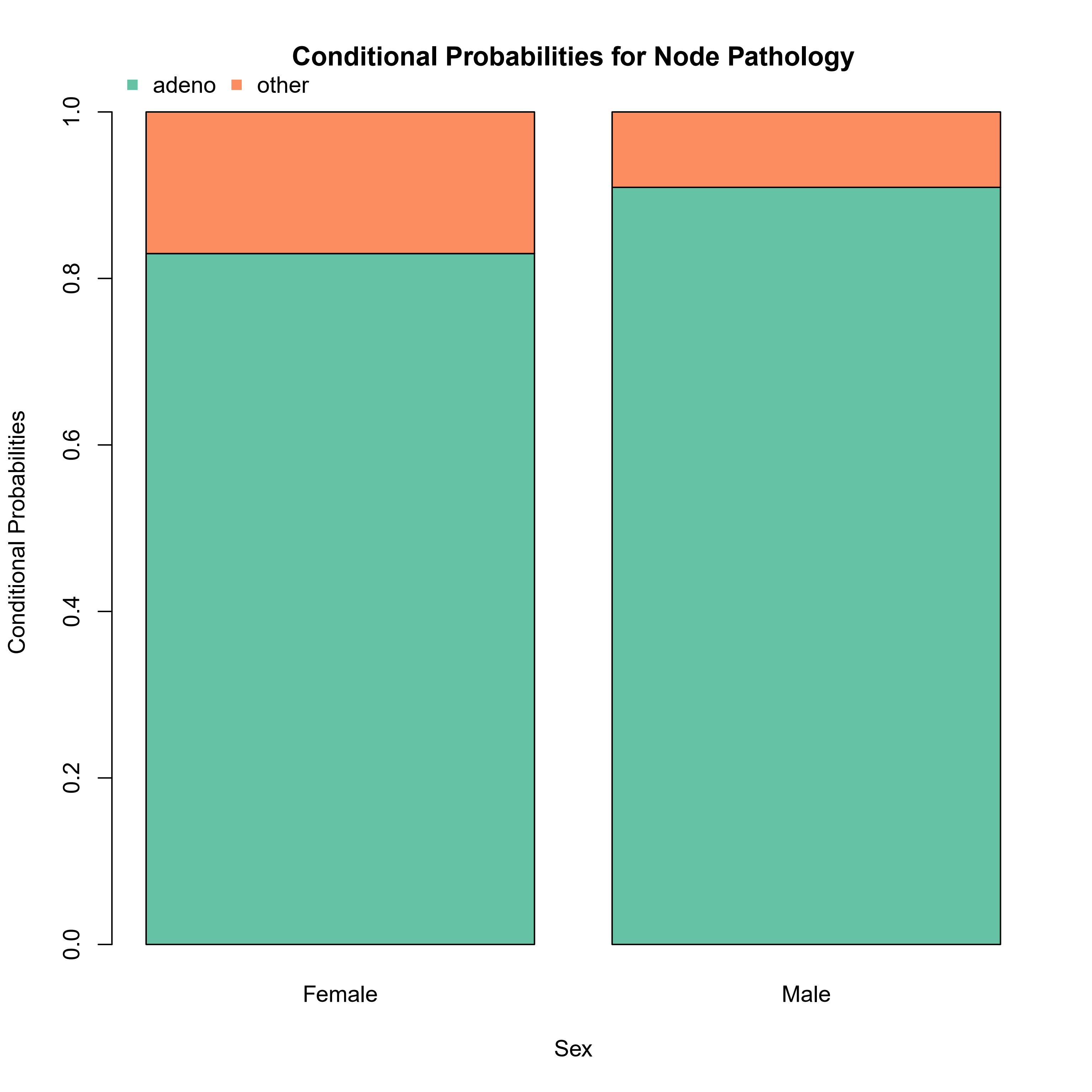


Supplementary figure 4. Conditional probability distribution of node pathology given the different levels of its parent (Sex)


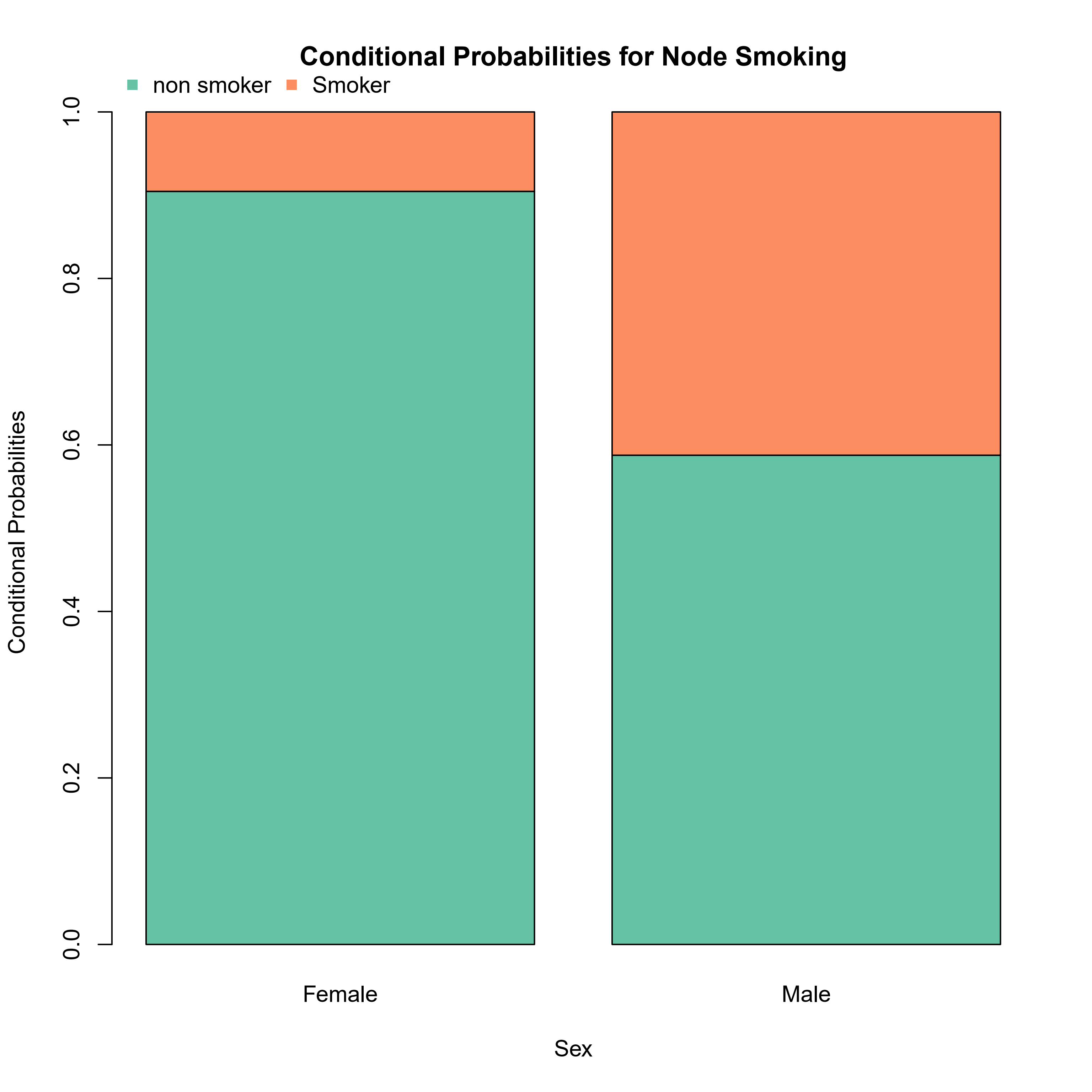


Supplementary figure 5. Conditional probability distribution of node smoking given the different levels of its parent (Sex)


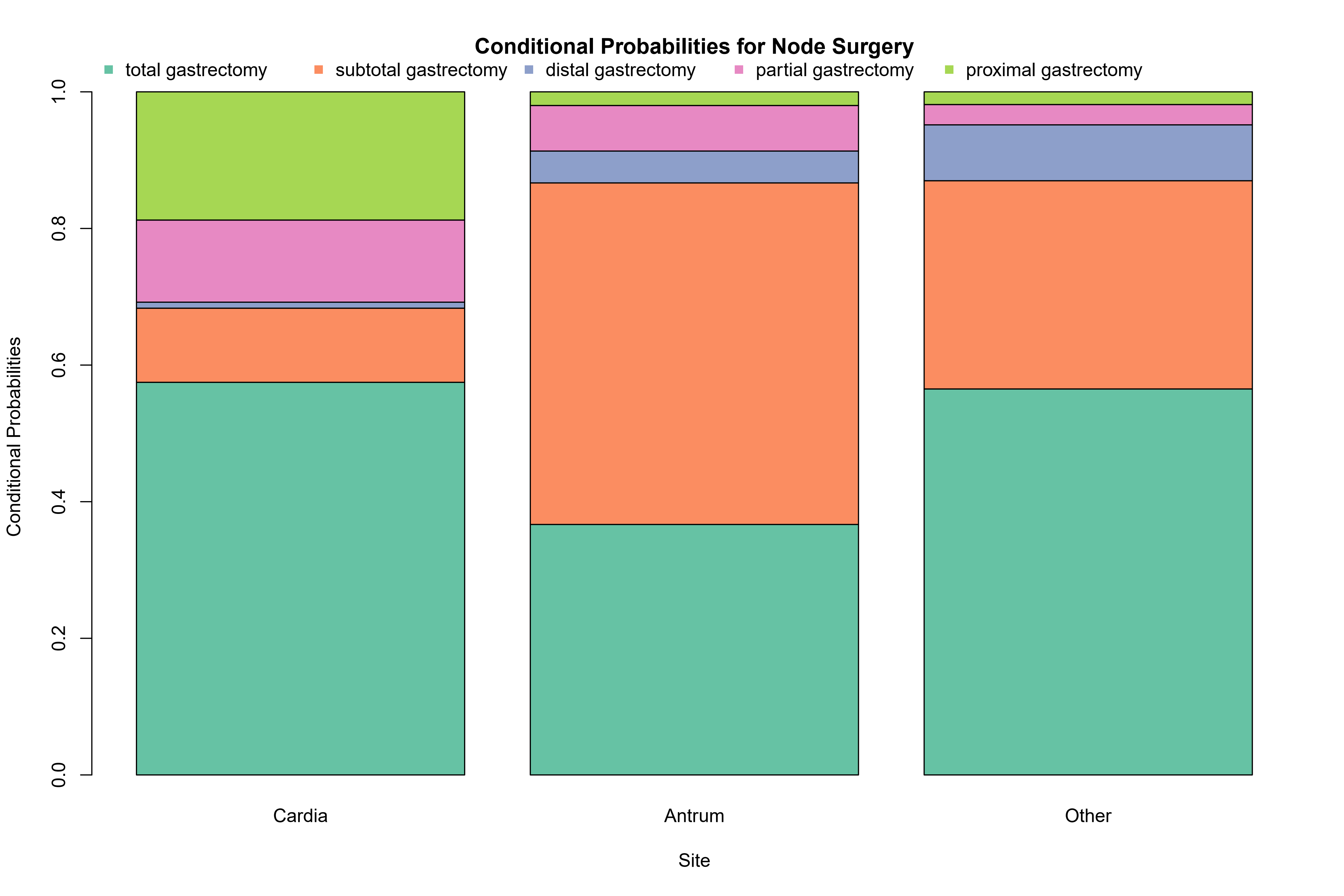


Supplementary figure 6. Conditional probability distribution of node surgery given the different levels of its parent (Site)
